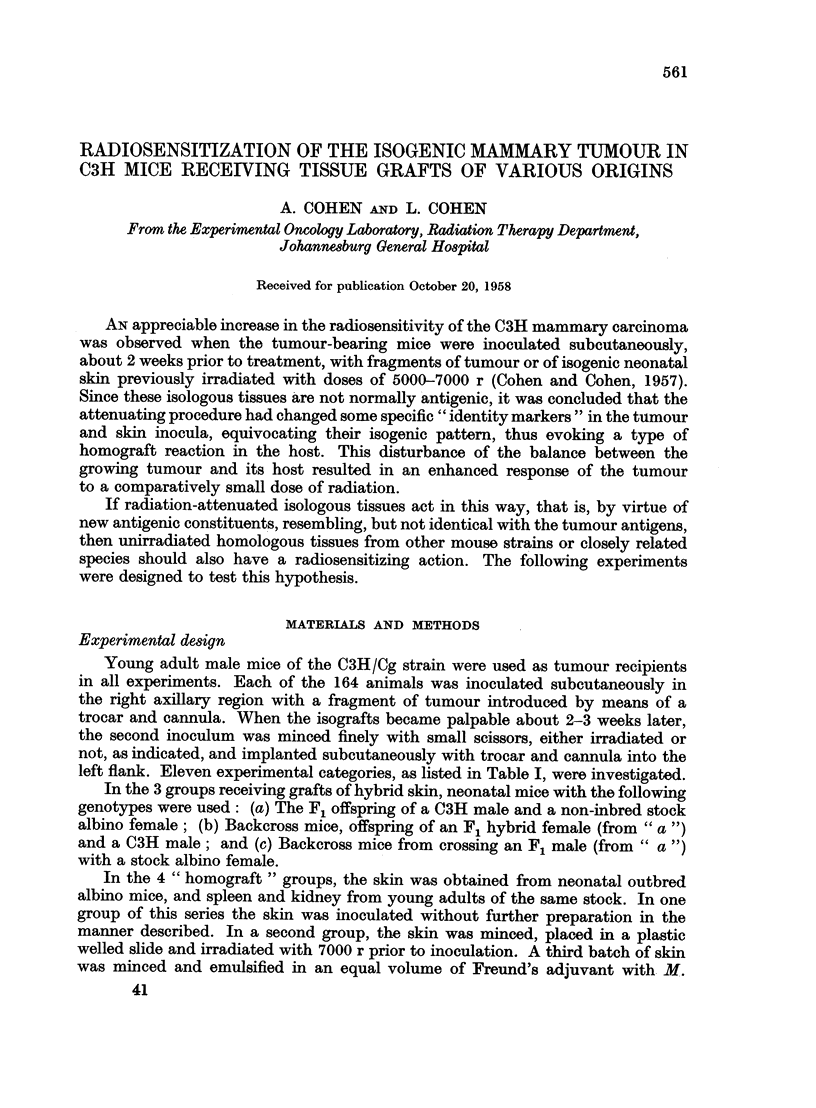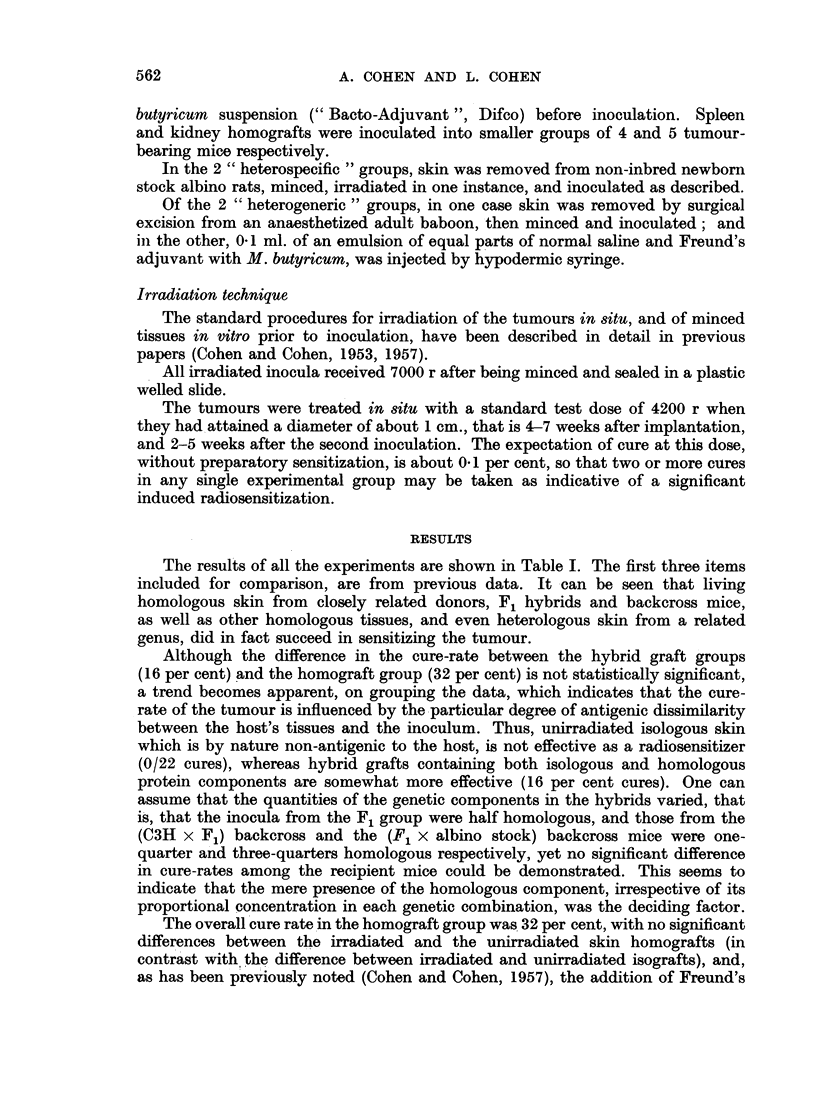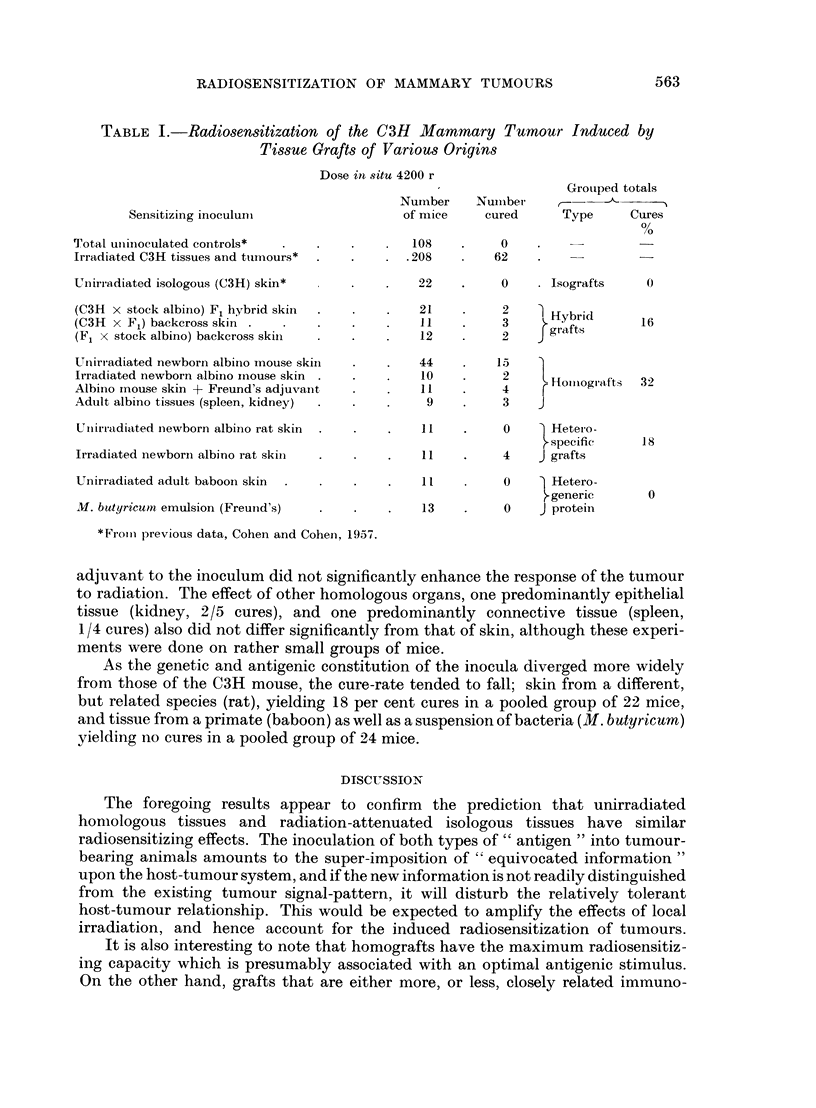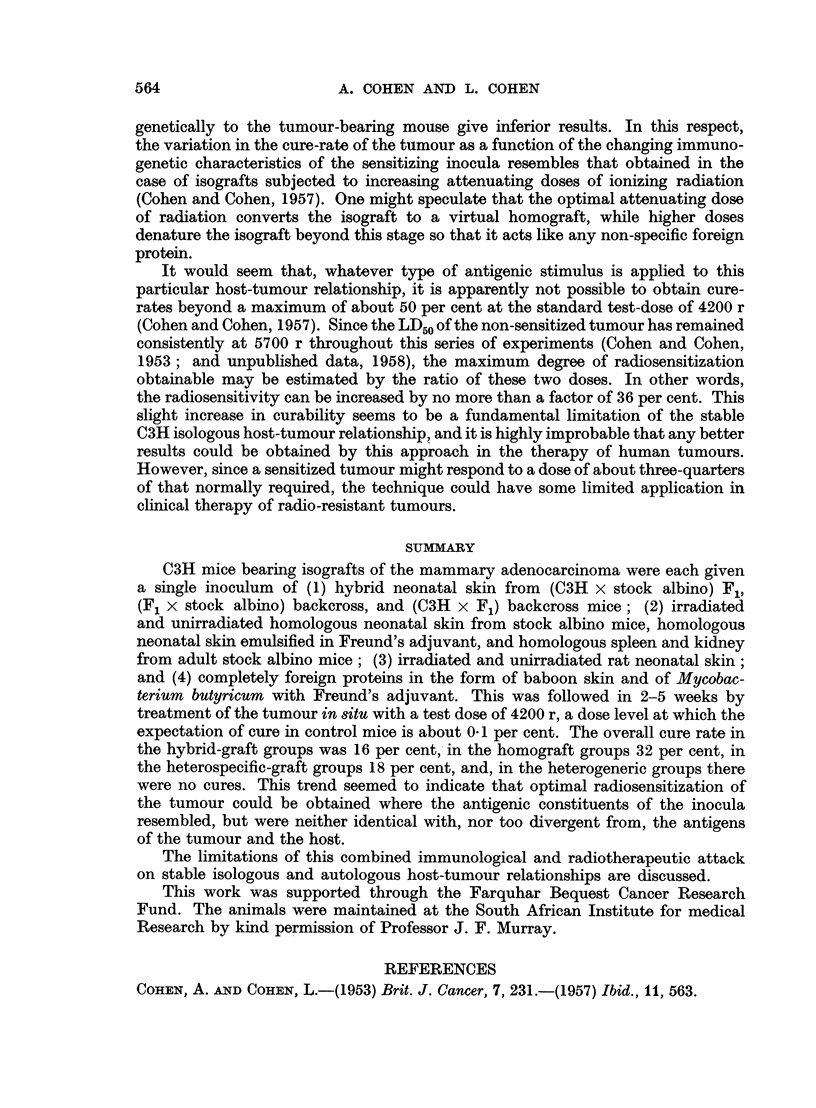# Radiosensitization of the Isogenic Mammary Tumour in C3H Mice Receiving Tissue Grafts of Various Origins

**DOI:** 10.1038/bjc.1958.65

**Published:** 1958-12

**Authors:** A. Cohen, L. Cohen


### RADIOSENSITIZATION OF THE ISOGENIC MAMMARY TUMOUR IN C3H MICE RECEIVING TISSUE GRAFTS OF VARIOUS ORIGINS

A. COHEN AND L. COHEN

*From the Experimental Oncology Laboratory, Radiation Therapy Department, Johannesburg General Hospital*

Received for publication October 20, 1958

AN appreciable increase in the radiosensitivity of the C3H mammary carcinoma was observed when the tumour-bearing mice were inoculated subcutaneously, about 2 weeks prior to treatment, with fragments of tumour or of isogenic neonatal skin previously irradiated with doses of 5000–7000 r (Cohen and Cohen, 1957). Since these isologous tissues are not normally antigenic, it was concluded that the attenuating procedure had changed some specific " identity markers " in the tumour and skin inocula, equivocating their isogenic pattern, thus evoking a type of homograft reaction in the host. This disturbance of the balance between the growing tumour and its host resulted in an enhanced response of the tumour to a comparatively small dose of radiation.

If radiation-attenuated isologous tissues act in this way, that is, by virtue of new antigenic constituents, resembling, but not identical with the tumour antigens, then unirradiated homologous tissues from other mouse strains or closely related species should also have a radiosensitizing action. The following experiments were designed to test this hypothesis.

### MATERIALS AND METHODS

### *Experimental design*

Young adult male mice of the C3H/Cg strain were used as tumour recipients in all experiments. Each of the 164 animals was inoculated subcutaneously in the right axillary region with a fragment of tumour introduced by means of a trocar and cannula. When the isografts became palpable about 2–3 weeks later, the second inoculum was minced finely with small scissors, either irradiated or not, as indicated, and implanted subcutaneously with trocar and cannula into the left flank. Eleven experimental categories, as listed in Table I, were investigated.

In the 3 groups receiving grafts of hybrid skin, neonatal mice with the following genotypes were used : (*a*) The $F_1$ offspring of a C3H male and a non-inbred stock albino female ; (b) Backcross mice, offspring of an $F_1$ hybrid female (from " *a* ") and a C3H male ; and (*c*) Backcross mice from crossing an $F_1$ male (from " *a* ") with a stock albino female.

In the 4 " homograft " groups, the skin was obtained from neonatal outbred albino mice, and spleen and kidney from young adults of the same stock. In one group of this series the skin was inoculated without further preparation in the manner described. In a second group, the skin was minced, placed in a plastic welled slide and irradiated with 7000 r prior to inoculation. A third batch of skin was minced and emulsified in an equal volume of Freund's adjuvant with *M.*

*butyricum* suspension (" Bacto-Adjuvant ", Difco) before inoculation. Spleen and kidney homografts were inoculated into smaller groups of 4 and 5 tumour-bearing mice respectively.

In the 2 " heterospecific " groups, skin was removed from non-inbred newborn stock albino rats, minced, irradiated in one instance, and inoculated as described.

Of the 2 " heterogeneric " groups, in one case skin was removed by surgical excision from an anaesthetized adult baboon, then minced and inoculated ; and in the other, 0·1 ml. of an emulsion of equal parts of normal saline and Freund's adjuvant with *M. butyricum*, was injected by hypodermic syringe.

*Irradiation technique*

The standard procedures for irradiation of the tumours *in situ*, and of minced tissues *in vitro* prior to inoculation, have been described in detail in previous papers (Cohen and Cohen, 1953, 1957).

All irradiated inocula received 7000 r after being minced and sealed in a plastic welled slide.

The tumours were treated *in situ* with a standard test dose of 4200 r when they had attained a diameter of about 1 cm., that is 4–7 weeks after implantation, and 2–5 weeks after the second inoculation. The expectation of cure at this dose, without preparatory sensitization, is about 0·1 per cent, so that two or more cures in any single experimental group may be taken as indicative of a significant induced radiosensitization.

### RESULTS

The results of all the experiments are shown in Table I. The first three items included for comparison, are from previous data. It can be seen that living homologous skin from closely related donors, $F_1$ hybrids and backcross mice, as well as other homologous tissues, and even heterologous skin from a related genus, did in fact succeed in sensitizing the tumour.

Although the difference in the cure-rate between the hybrid graft groups (16 per cent) and the homograft group (32 per cent) is not statistically significant, a trend becomes apparent, on grouping the data, which indicates that the cure-rate of the tumour is influenced by the particular degree of antigenic dissimilarity between the host's tissues and the inoculum. Thus, unirradiated isologous skin which is by nature non-antigenic to the host, is not effective as a radiosensitizer (0/22 cures), whereas hybrid grafts containing both isologous and homologous protein components are somewhat more effective (16 per cent cures). One can assume that the quantities of the genetic components in the hybrids varied, that is, that the inocula from the $F_1$ group were half homologous, and those from the (C3H $\times$ $F_1$) backcross and the ($F_1$ $\times$ albino stock) backcross mice were one-quarter and three-quarters homologous respectively, yet no significant difference in cure-rates among the recipient mice could be demonstrated. This seems to indicate that the mere presence of the homologous component, irrespective of its proportional concentration in each genetic combination, was the deciding factor.

The overall cure rate in the homograft group was 32 per cent, with no significant differences between the irradiated and the unirradiated skin homografts (in contrast with the difference between irradiated and unirradiated isografts), and, as has been previously noted (Cohen and Cohen, 1957), the addition of Freund's

TABLE I.—*Radiosensitization of the C3H Mammary Tumour Induced by Tissue Grafts of Various Origins*

Dose *in situ* 4200 r

| Sensitizing inoculum | Number of mice | Number cured | Grouped totals Type | Cures % |
|---|---|---|---|---|
| Total uninoculated controls* | 108 | 0 | — | — |
| Irradiated C3H tissues and tumours* | 208 | 62 | — | — |
| Unirradiated isologous (C3H) skin* | 22 | 0 | Isografts | 0 |
| (C3H × stock albino) $F_1$ hybrid skin | 21 | 2 | Hybrid grafts | 16 |
| (C3H × $F_1$) backcross skin | 11 | 3 | | |
| ($F_1$ × stock albino) backcross skin | 12 | 2 | | |
| Unirradiated newborn albino mouse skin | 44 | 15 | Homografts | 32 |
| Irradiated newborn albino mouse skin | 10 | 2 | | |
| Albino mouse skin + Freund's adjuvant | 11 | 4 | | |
| Adult albino tissues (spleen, kidney) | 9 | 3 | | |
| Unirradiated newborn albino rat skin | 11 | 0 | Hetero-specific grafts | 18 |
| Irradiated newborn albino rat skin | 11 | 4 | | |
| Unirradiated adult baboon skin | 11 | 0 | Hetero-generic protein | 0 |
| *M. butyricum* emulsion (Freund's) | 13 | 0 | | |

*From previous data, Cohen and Cohen, 1957.

adjuvant to the inoculum did not significantly enhance the response of the tumour to radiation. The effect of other homologous organs, one predominantly epithelial tissue (kidney, 2/5 cures), and one predominantly connective tissue (spleen, 1/4 cures) also did not differ significantly from that of skin, although these experiments were done on rather small groups of mice.

As the genetic and antigenic constitution of the inocula diverged more widely from those of the C3H mouse, the cure-rate tended to fall; skin from a different, but related species (rat), yielding 18 per cent cures in a pooled group of 22 mice, and tissue from a primate (baboon) as well as a suspension of bacteria (*M. butyricum*) yielding no cures in a pooled group of 24 mice.

### DISCUSSION

The foregoing results appear to confirm the prediction that unirradiated homologous tissues and radiation-attenuated isologous tissues have similar radiosensitizing effects. The inoculation of both types of " antigen " into tumour-bearing animals amounts to the super-imposition of " equivocated information " upon the host-tumour system, and if the new information is not readily distinguished from the existing tumour signal-pattern, it will disturb the relatively tolerant host-tumour relationship. This would be expected to amplify the effects of local irradiation, and hence account for the induced radiosensitization of tumours.

It is also interesting to note that homografts have the maximum radiosensitizing capacity which is presumably associated with an optimal antigenic stimulus. On the other hand, grafts that are either more, or less, closely related immunogenetically to the tumour-bearing mouse give inferior results. In this respect, the variation in the cure-rate of the tumour as a function of the changing immunogenetic characteristics of the sensitizing inocula resembles that obtained in the case of isografts subjected to increasing attenuating doses of ionizing radiation (Cohen and Cohen, 1957). One might speculate that the optimal attenuating dose of radiation converts the isograft to a virtual homograft, while higher doses denature the isograft beyond this stage so that it acts like any non-specific foreign protein.

It would seem that, whatever type of antigenic stimulus is applied to this particular host-tumour relationship, it is apparently not possible to obtain cure-rates beyond a maximum of about 50 per cent at the standard test-dose of 4200 r (Cohen and Cohen, 1957). Since the $LD_{50}$ of the non-sensitized tumour has remained consistently at 5700 r throughout this series of experiments (Cohen and Cohen, 1953; and unpublished data, 1958), the maximum degree of radiosensitization obtainable may be estimated by the ratio of these two doses. In other words, the radiosensitivity can be increased by no more than a factor of 36 per cent. This slight increase in curability seems to be a fundamental limitation of the stable C3H isologous host-tumour relationship, and it is highly improbable that any better results could be obtained by this approach in the therapy of human tumours. However, since a sensitized tumour might respond to a dose of about three-quarters of that normally required, the technique could have some limited application in clinical therapy of radio-resistant tumours.

SUMMARY

C3H mice bearing isografts of the mammary adenocarcinoma were each given a single inoculum of (1) hybrid neonatal skin from (C3H × stock albino) $F_1$, ($F_1$ × stock albino) backcross, and (C3H × $F_1$) backcross mice; (2) irradiated and unirradiated homologous neonatal skin from stock albino mice, homologous neonatal skin emulsified in Freund's adjuvant, and homologous spleen and kidney from adult stock albino mice; (3) irradiated and unirradiated rat neonatal skin; and (4) completely foreign proteins in the form of baboon skin and of *Mycobacterium butyricum* with Freund's adjuvant. This was followed in 2–5 weeks by treatment of the tumour *in situ* with a test dose of 4200 r, a dose level at which the expectation of cure in control mice is about 0·1 per cent. The overall cure rate in the hybrid-graft groups was 16 per cent, in the homograft groups 32 per cent, in the heterospecific-graft groups 18 per cent, and, in the heterogeneric groups there were no cures. This trend seemed to indicate that optimal radiosensitization of the tumour could be obtained where the antigenic constituents of the inocula resembled, but were neither identical with, nor too divergent from, the antigens of the tumour and the host.

The limitations of this combined immunological and radiotherapeutic attack on stable isologous and autologous host-tumour relationships are discussed.

This work was supported through the Farquhar Bequest Cancer Research Fund. The animals were maintained at the South African Institute for medical Research by kind permission of Professor J. F. Murray.

### REFERENCES

COHEN, A. AND COHEN, L.—(1953) *Brit. J. Cancer*, **7**, 231.—(1957) *Ibid.*, **11**, 563.